# Type VI secretion system sheath inter‐subunit interactions modulate its contraction

**DOI:** 10.15252/embr.201744416

**Published:** 2017-12-08

**Authors:** Maximilian Brackmann, Jing Wang, Marek Basler

**Affiliations:** ^1^ Focal Area Infection Biology, Biozentrum University of Basel Basel Switzerland

**Keywords:** contractile tails, microbiology, phages, type VI secretion system, Microbiology, Virology & Host Pathogen Interaction, Structural Biology

## Abstract

Secretion systems are essential for bacteria to survive and manipulate their environment. The bacterial type VI secretion system (T6SS) generates the force needed for protein translocation by the contraction of a long polymer called sheath. The sheath is a six‐start helical assembly of interconnected VipA/VipB subunits. The mechanism of T6SS sheath contraction is unknown. Here, we show that elongating the N‐terminal VipA linker or eliminating charge of a specific VipB residue abolishes sheath contraction and delivery of effectors into target cells. Mass spectrometry analysis identified the inner tube protein Hcp, spike protein VgrG, and other components of the T6SS baseplate significantly enriched in samples of the stable non‐contractile sheaths. The ability to lock the T6SS in the pre‐firing state opens new possibilities for understanding its mode of action.

## Introduction

Bacteria have evolved various protein nanomachines to translocate macromolecules across biological membranes. A subset of these nanomachines is composed of a rigid tube surrounded by a contractile sheath, which is attached to a baseplate. The sheath is initially assembled in a high‐energy, extended state and then quickly transits to a low‐energy, contracted state. The sheath contraction pushes the inner tube, through a membrane complex into the target cell, where it delivers its cargo. The bacterial type VI secretion system (T6SS) uses this mechanism to deliver proteins across membranes 1, 2, 3, 4, 5.

Biogenesis of the T6SS starts by formation of an integral membrane complex of TssJ, TssL, and TssM on which the baseplate assembles 6, 7. TssE, TssF, TssG, and TssK constitute the baseplate with a trimer of the spike protein VgrG in the center 6, 8. Baseplate assembly is required to initiate polymerization of the hexameric tube protein Hcp. The tube serves as a template for rapid polymerization of the extended sheath composed of heterodimeric protomers of VipA (TssB) and VipB (TssC) 9, 10, 11, 12, 13. In addition, two different classes of TssA proteins are important for the initiation of T6SS sheath assembly and its elongation 14, 15, which progresses throughout the whole cell and thus allows the use of live‐cell fluorescence microscopy to monitor sheath dynamics 1, 7, 16, 17, 18, 19. Contraction of the T6SS sheath pushes the spike and the inner tube out of the cell into a neighboring cell, which leads to the delivery of effectors that are associated with the spike or the inner tube 20, 21, 22. Among substrates of the T6SS are anti‐eukaryotic as well as anti‐bacterial effectors that are used for host interaction and interbacterial competition 23, 24, 25.

The sheath can be described as a six‐start helix and thus with six strands or as a stack of rings composed of six VipA/VipB heterodimeric subunits, which are interconnected by N‐ and C‐terminal linkers in the inner domain of the sheath 26, 27. Inner domains of sheaths of contractile phage, R‐type pyocin, and T6SS likely maintain the connectivity of the sheath during contraction and are evolutionarily related to each other; however, the surface‐exposed domains are distinct 12, 26, 27, 28, 29, 30, 31, 32. Specifically, T6SS sheath contains a surface‐exposed Domain 3 that plays a crucial role in sheath recycling 26, 27, 31, 32. The mechanism of T6SS sheath contraction is largely unknown because the sheath contracts during isolation from cells, and thus, characterization of the extended sheath was not possible until recently 1, 32.

Here, we identified two structural features of the T6SS sheath that play a critical role in its contraction. Using live‐cell fluorescence microscopy, we show that a single negatively charged residue located on the surface of the middle domain of the T6SS sheath is required for sheath contraction but not sheath assembly. We further show that the VipA N‐terminal linker is critical for sheath contraction. Insertion of two and more amino acid residues into this linker completely abrogated contraction and allowed us to isolate non‐contractile sheaths from cells for mass spectrometry and electron microscopy analysis. This analysis revealed that non‐contracted sheaths are stably associated with the inner Hcp tube and components of the T6SS baseplate. Overall, comparison of T6SS sheaths of *Francisella novicida* and *Vibrio cholerae* and the sheath of R‐type pyocins shows that conserved structural features are involved in sheath contraction and provides insights into T6SS assembly and its mode of action.

## Results and Discussion

### VipB residue D333 is important for contraction *in vivo*

Interactions of charged residues were previously suggested to be important for contraction of T4 phage sheath and R‐type pyocin sheath 28, 30. Analysis of interfaces that are expected to be present only in the contracted form of the T6SS sheath of *V. cholerae* suggested that VipB residues K223 and D333 located on two different VipB–VipB interfaces, one between protomers of a single sheath strand and the second between protomers on two adjacent strands, significantly contribute to the stability of the contracted structure 27. This suggests that the energy released by the formation of these interactions could contribute to the sheath contraction.

**Figure 1:**
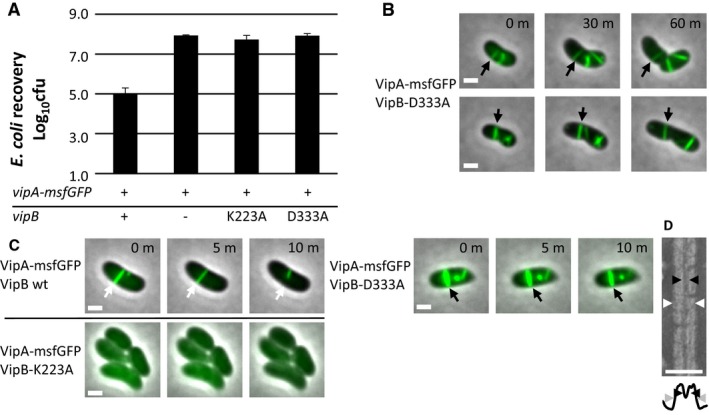
Charged residues of VipB are essential for sheath assembly and contraction *Escherichia coli* survival (± SD, *N* = 3) after 3‐h competition with indicated *Vibrio cholerae* strains in a 1:10 ratio on plate; the first column is significantly different from the rest (*P* < 0.01).One‐hour timelapses of *V. cholerae vipA‐msfGFP, vipB* ^*−*^ complemented with *vipB‐*D333A on pBAD24. Two examples of stable T6SS sheaths are highlighted with arrows. Images are composite images of phase contrast and fluorescence channels. Scale bars are 1 μm.Ten‐minute timelapse images of *V. cholerae vipA‐msfGFP, vipB* ^*−*^ complemented with *vipB* wild type, *vipB*‐K223A, or *vipB*‐D333A on pBAD24. A dynamic, contracting (VipB wild type, white arrow) and a stable, static (VipB‐D333A, black arrow) sheath are highlighted with arrows. VipB‐K223A does not assemble T6SS sheaths. Images are composite images of phase contrast and fluorescence channels. Scale bars are 1 μm.Crop of an electron micrograph of purified VipB‐D333A sheath and below a plot of the summed intensities. The inner diameter is marked with black arrowheads and the outer diameter with white or gray arrowheads. Scale bar is 50 nm.

To test this hypothesis, we mutated K223 and D333 to alanine and expressed the mutated *vipB* in a *vipA‐msfGFP*,* ΔvipB* background to allow monitoring of sheath dynamics in live cells. The K223A mutation impaired assembly of the sheath, and no elongated sheaths were observed (Fig 1C and Movie EV1). This was also reflected in the inability of the VipB‐K223A mutant to kill target cells (Fig 1A). Interestingly, the VipB‐D333A mutant assembled into non‐dynamic sheath structures that were stable *in vivo* over more than one hour of imaging (Fig 1B and Movie EV1 and EV2). The number of sheath structures per cell was comparable to the number of structures assembled in wild‐type cells suggesting that D333 residue is not critical for sheath assembly. Similarly to the extended wild‐type sheaths, the VipB‐D333A sheaths were stable even in the presence of ClpV. Importantly, the VipB‐D333A mutation also completely blocked target cell killing (Fig 1A).

### VipA linker is critical for sheath contraction *in vivo*

**Figure EV1:**
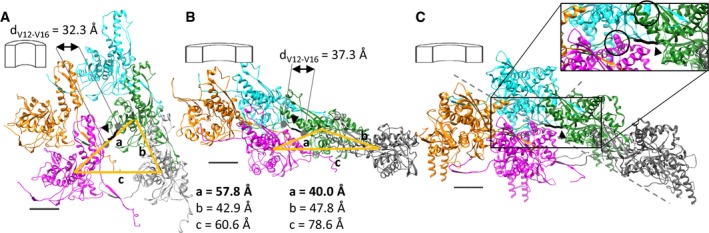
Protomers of contractile sheaths are interconnected by linkers that are stretched in the contracted state Structure of an R‐type pyocin sheath in the extended state (PDB ID: 3J9Q) viewed from inside the sheath. Individual protomers are interlaced via linkers (black, arrowhead) through interactions formed by β‐sheets.Structure of an R‐type pyocin sheath in the contracted state (PDB ID: 3J9R). The linkers interconnecting (black, arrowhead) two strands are stretched.Structure of the contracted T6SS sheath from *V. cholerae* (PDB ID: 3J9G). The backbone of the linker region of VipA that connects the green protomer with the magenta protomer is shown thick in black (arrowhead). Mutated residues on VipB are highlighted in black circles in the inset. D333 is shown on the cyan protomer, and K223 is shown on the magenta protomer. Distances between center of masses of different protomers are depicted as orange lines.Data information: The scale bars in all panels are 20 Å.

Recent atomic models of T6SS sheaths in a contracted state and structures of the R‐type pyocin in an extended and contracted state identified intermolecular linkers important for sheath function 26, 27, 30. Interestingly, the N‐terminal linker of the R‐type pyocin sheath appears more stretched in the contracted sheath than in the extended sheath (Fig EV1). We hypothesized that stretching of the N‐terminal sheath linker of a basal ring upon contraction results in pulling on the VipA N‐terminal linker of the next sheath ring, which in turn triggers its contraction. Such a mechanism would lead to propagation of contraction through the whole sheath, as suggested earlier for the T4 phage sheath 33. We decided to test this hypothesis by generating a series of mutant *V. cholerae* T6SS sheaths with longer VipA linkers. We inserted 1–7 amino acids of the native “AEVELPL” sequence of the linker after residue 25 of VipA wild‐type protein (labeled here as VipA‐N1 to VipA‐N7). To monitor the assembly and contraction, we fused VipA and its variants to msfGFP and expressed it from pBAD24 plasmid in the absence of chromosomal *vipA* 1, 27.

**Figure 2:**
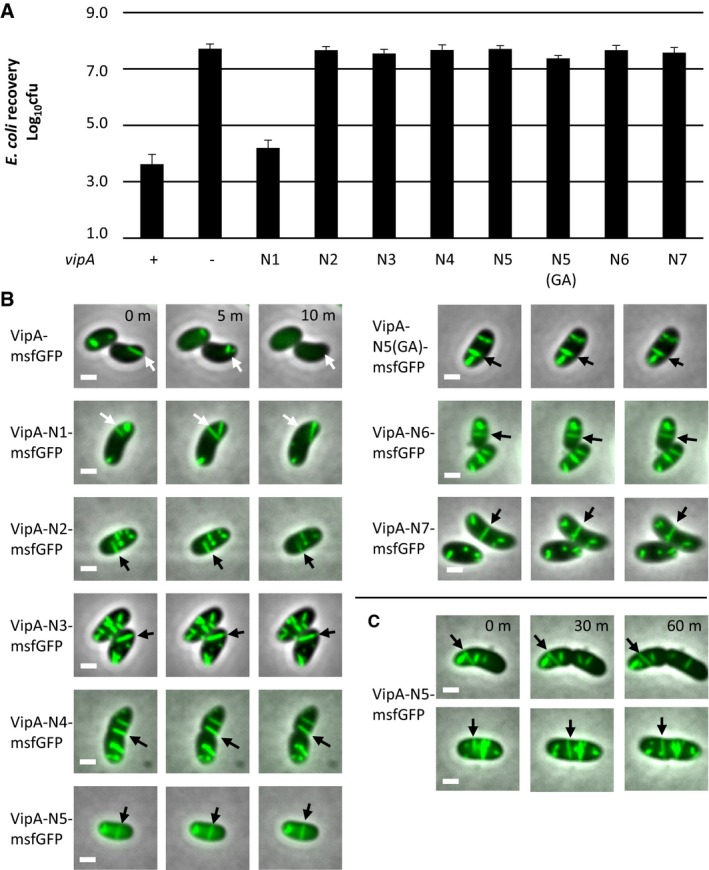
Length of N‐terminal linker of VipA controls sheath contraction *Escherichia coli* survival (± SD, *N* = 3) after 3‐h competition with indicated *Vibrio cholerae* strains in a 1:10 ratio on plate; the first and third columns are significantly different from the rest (*P* < 0.01).Fluorescence timelapse images of *V. cholerae vipA* ^*−*^ complemented with indicated msfGFP‐tagged‐*vipA* variants on pBAD24. Arrows highlight sheaths that contract (wild type, N1, white arrows) or are static (N2 or higher, black arrows). Images are composite images of phase contrast and fluorescence channels.Long timelapse of *V. cholerae vipA* ^*−*^ complemented with *vipA‐N5‐msfGFP* on pBAD24. Images are composite images of phase contrast and fluorescence channels.Data information: Scale bars are 1 μm.

All mutant T6SS sheaths assembled with a frequency similar to that of the wild‐type sheath (Fig 2B); however, the frequency of sheath contraction was strongly dependent on the linker length (Fig 2B, Movie EV3). Whereas insertion of one amino acid (VipA‐N1) had almost no effect on sheath dynamics, an elongation by two or more amino acids (VipA‐N2‐7) reduced the fraction of sheaths that contract within 5 min from 50% (of 159 VipA wild‐type structures counted, 85 contracted) to 0% (of 204 VipA‐N3 structures counted, none contracted). Many of these mutant sheaths were stable over one hour of imaging (Fig 2C and Movie EV4). Sheaths that occasionally broke after extensive bending caused by the movement and growth of cells were however quickly disassembled (Movie EV4). VipA with “AGAGA” sequence inserted, labeled as VipA‐N5(GA), also assembled stable full‐length sheaths (Fig 2B, Movie EV3), suggesting that VipA linker length, but not its sequence, is specifically critical for sheath stability. Furthermore, the killing of *Escherichia coli* MG1655 by *V. cholerae* T6SS (Fig 2A) was strongly dependent on the length of the VipA linker. Whereas an extension of this linker by one amino acid had no effect on the killing efficiency, an elongation by two amino acids completely abolished the killing of target cells.

The length of the sheaths *in vivo* did not differ between wild‐type and the non‐contractile sheaths VipB‐D333A or VipA‐N3. The mean length of sheaths was between 0.53 μm and 0.59 μm for all measured mutants (Table EV1). This suggests that the length of sheaths is limited by the size of the cell and the orientation of the T6SS inside cells as shown previously 19 rather than by the mutations that were introduced.

### Stable sheaths assemble from baseplate and around Hcp

To analyze the stable mutant sheaths in more detail, we isolated them by using an approach similar to the one used for the isolation of the wild‐type contracted sheath 1, 27. Mutant sheaths were expressed in a non‐flagellated *V. cholerae* strain, cells were lysed, and sheaths were purified from soluble proteins and cell debris using ultra‐centrifugation. The isolated sheaths were analyzed by negative staining electron microscopy.

**Figure 3:**
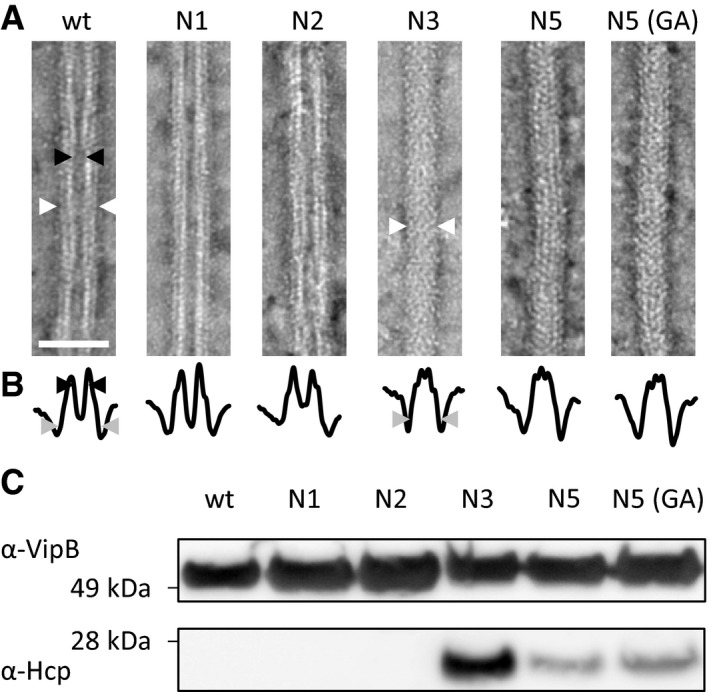
Hcp is enriched in VipA mutants with a linker elongated by three or more amino acids Electron micrographs of isolated sheath samples. Wild‐type sheath and those with additional one or two amino acids inserted are hollow but with three or more amino acids show a protein density in the center. Black arrowheads mark the inner diameter, and white arrowheads mark the outer diameter. Arrowheads are shown for one example of the two different types of structures. Scale bar is 50 nm.Plot of summed intensities of the micrographs in (A). The inner diameter is marked with black arrowheads and the outer diameter with gray arrowheads. Arrowheads are shown for one example of the two different types of structures.Immunoblots against Hcp and VipB of the samples in (A). Source data are available online for this figure.

**Figure EV2:**
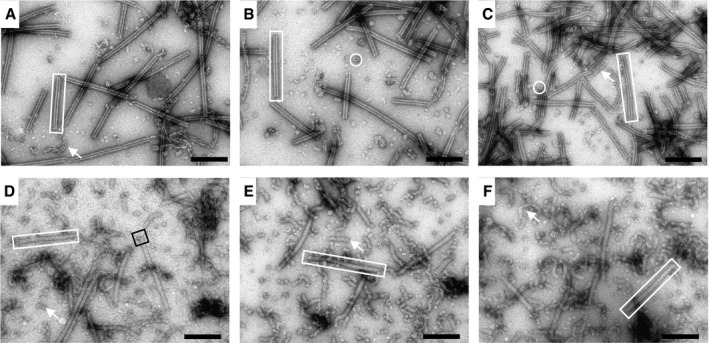
Isolated sheaths appear in two different conformations Negative‐stain electron micrographs of purified sheaths. 
A–F(A) Isolated wild‐type sheaths. (B) Isolated VipA‐N1 sheaths. (C) Isolated VipA‐N2 sheaths. (D) Isolated VipA‐N3 sheaths. (E) Isolated VipA‐N5 sheaths. (F) Isolated VipA‐N5 (GA) sheaths. Hollow (A–C) and filled sheaths (D–F) can be observed (rectangles) as well as cogwheel‐like structures (circles). Disintegrated sheaths (arrows) are observed in some cases, especially for N3, N5, and N5(GA). An example of a baseplate‐like structure (black square) is highlighted. Scale bars are 200 nm.

Analysis of VipB‐D333A sheath sample revealed partially fragmented hollow structures with an outer diameter of 260 nm, thus resembling contracted sheaths (Fig 1D). This suggests that during isolation, the VipB‐D333A sheaths contract and the D333A mutation destabilizes the contracted structure, which leads to partial fragmentation. Similarly, VipA‐N2 sheaths closely resembled the wild‐type contracted sheaths as they appeared hollow, had the inner diameter of 100 Å, and the outer diameter of 260 Å (Figs 3A and B, and EV2). Interestingly, VipA‐N3, VipA‐N5, and VipA‐N5(GA) mutant sheath diameters were ≈200 Å and thus narrower than wild type (Figs 3A and B, and EV2). Importantly, uranyl acetate stain was clearly unable to penetrate the sheaths, suggesting that VipA‐N3, VipA‐N5, and VipA‐N5(GA) mutant sheaths were filled with additional proteins (Fig 3A and B).

**Figure EV3:**
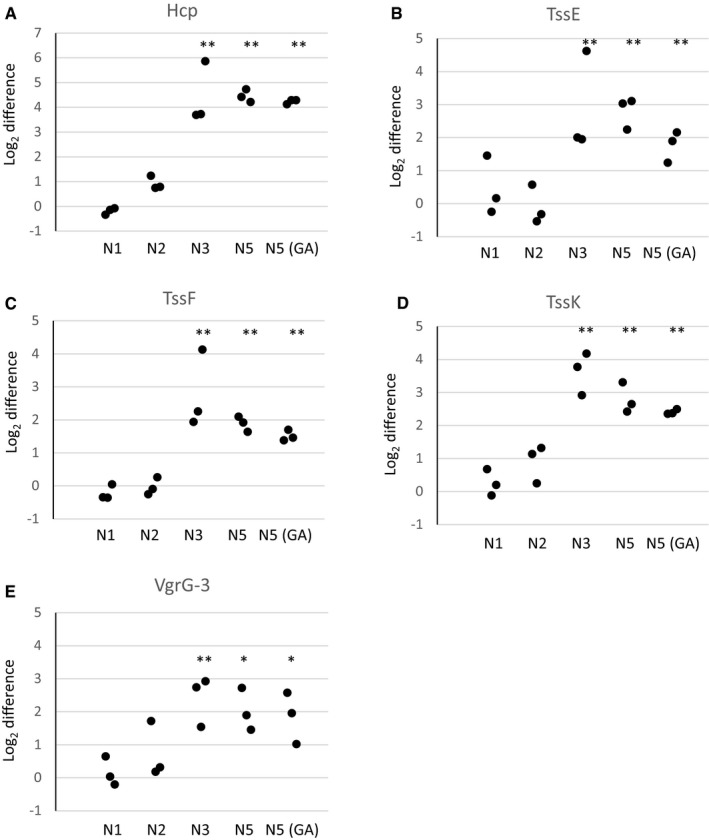
The tube protein Hcp, baseplate proteins TssE, TssF, and TssK, and VgrG‐3 are enriched in VipA mutants with a linker elongated by three or more amino acids A–EMS1 peak intensities of different T6SS‐related proteins are displayed relative to wild‐type protein levels. Three independent biological replicates were analyzed, and individual data points are displayed. (A) Hcp. (B) TssE. (C) TssF. (D) TssK. (E) VgrG‐3. *P ≤ 0.02, **P ≤ 0.001 (Student's *t*‐test).

To identify the proteins that were associated with the mutant sheaths, we subjected the VipA wild‐type, N1, 2, 3, 5, and 5(GA) samples to mass spectrometry analysis (Table EV2 and Dataset EV1). Besides VipA and VipB proteins, VipA‐N3, VipA‐N5, and VipA‐N5(GA) sheath preparations contained large amounts of Hcp, as further confirmed by Western blot (Fig 3C, lower panel). The presence of Hcp in these mutant sheath samples explains the solid appearance on negative‐stain EM (Fig 3A). Altogether, 10 of 16 T6SS‐related proteins were identified in all independently purified triplicates. Interestingly, six of these proteins were specifically enriched in samples with a linker elongated by three or more amino acids (*P* < 0.02). Among the enriched proteins are baseplate proteins TssE, TssF, TssG, and TssK but also the tip component VgrG‐3 (Fig EV3). VgrG‐1 and VgrG‐2 as well as most other proteins of the membrane complex have not been identified in all replicates and thus were excluded from the analysis.

**Figure EV4:**
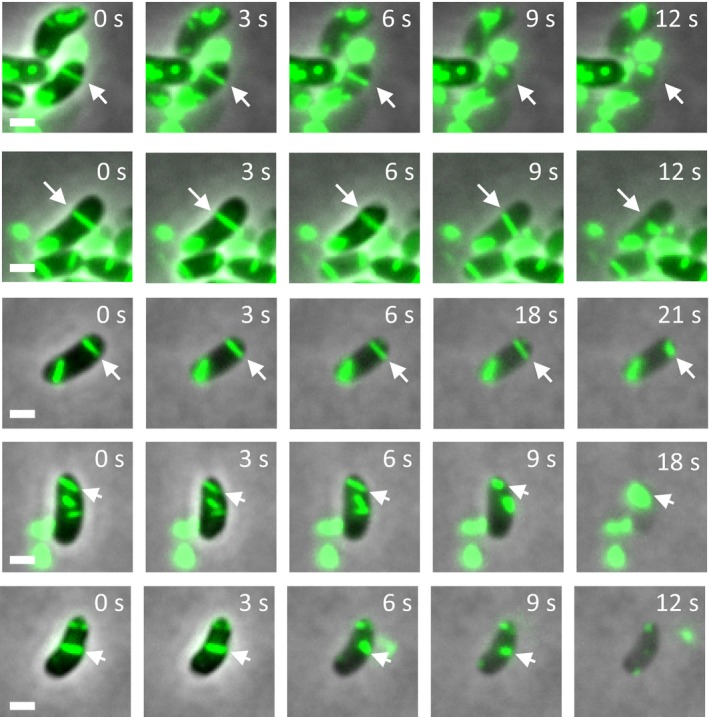
VipA‐N2 sheaths contract during cell lysis *Vibrio cholerae vipA* ^*−*^ complemented with msfGFP‐tagged *vipA‐N2* on pBAD24 are incubated on an agarose pad containing CelLytic B and EDTA. Lysis happens within minutes, and arrows mark sheaths that contract immediately before, during, or shortly after lysis. Representative images are shown. Scale bars are 1 μm.

Interestingly, VipA‐N2 sheaths are stable in the cell cytosol in the presence of ClpV, however, can only be isolated in a conformation closely resembling contracted sheath. To test whether VipA‐N2 sheaths contract during isolation, we imaged the mutant sheaths on an agarose pad containing low concentrations of CelLytic B and EDTA, the lytic agents that we use for bacterial cell lysis. We found that VipA‐N2 sheaths shorten their length by ≈50% shortly before, during, or immediately after lysis, which indicates that VipA‐N2 sheaths indeed assemble in an extended state but may contract during cell lysis (Fig EV4 and Movie EV5).

### Mutant sheaths assemble only from a functional baseplate and the non‐contractile phenotype is dominant

**Figure 4:**
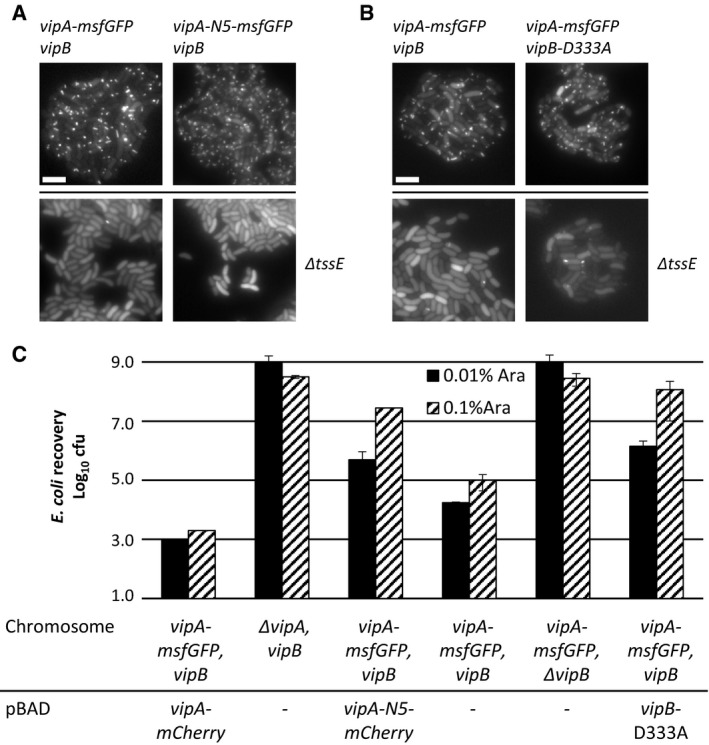
Assembly of non‐contractile sheaths depends on the presence of TssE and mutant sheath subunits are dominant Fluorescence microscopy images of *Vibrio cholerae vipA* ^*−*^ (upper panels) or *vipA* ^*−*^ *, tssE* ^*−*^ (lower panels) complemented with *vipA‐msfGFP* (left panels) or *vipA‐N5‐msfGFP* (right panels) on pBAD24.Fluorescence microscopy images of *V. cholerae vipA* ^*−*^ *, vipB* ^*−*^ (upper panels) or *vipA* ^*−*^ *, vipB* ^*−*^ *, tssE* ^*−*^ (lower panels) complemented with *vipA‐msfGFP* on pBAD24 and *vipB* (left panels) or *vipB‐*D333A (right panels) on pBAD33.
*Escherichia coli* survival (± SD, *N* = 2) after 3‐h competition with indicated *V. cholerae* strains in a 1:10 ratio on plates at two different arabinose concentrations.Data information: Scale bars are 5 μm.

To exclude the possibility that the non‐contractile sheaths are aberrant polymers that assemble independently of other T6SS components, we imaged their assembly in a strain that lacks the baseplate component TssE. In agreement with the previous observations that TssE is required for efficient sheath assembly 1, 34, the frequency of wild‐type T6SS sheath assembly decreased by 140‐fold in the absence of *tssE*. Similarly, the assembly of D333A or VipA‐N5 sheaths was clearly dependent on the presence of a functional baseplate since the number of structures assembled in the cells lacking *tssE* was reduced by at least 100‐fold (Fig 4A and B). This indicates that stable mutant sheaths assemble from a baseplate similarly to the wild‐type sheaths.

To test whether the mutant sheath subunits can block T6SS activity also in the presence of the wild‐type subunits, we induced expression of the VipA‐N5 mutant from pBAD24 plasmid in a strain expressing wild‐type sheath from the chromosome and measured the efficiency of *E. coli* killing. Low‐level induction of VipA‐N5 by 0.01% arabinose decreased the T6SS‐dependent killing of *E. coli* by 100‐fold. The T6SS activity was almost completely blocked by increasing the concentration of arabinose to 0.1% (Fig 4C) indicating that the ratio of wild‐type VipA to VipA‐N5 is important for effector delivery. No such inhibition was observed when wild‐type VipA was expressed from the plasmid (Fig 4C). Similarly, low‐level expression of VipB‐D333A decreased T6SS activity by 100‐fold and high level of expression blocked the T6SS activity completely (Fig 4C). This dominant negative phenotype suggests that the mutant subunits are structurally compatible with the wild‐type subunits, co‐assemble into the same structures, and thus block T6SS function.

### Concluding remarks

The T6SS is a highly dynamic system, and this complicates detailed biochemical and biophysical characterization of its mode of action. Here, we show that the system can be locked in the pre‐contraction state by mutagenesis of the linker connecting sheath subunits or changing interactions contributing to the stability of the contracted state. Importantly, some non‐contractile sheaths are stable during isolation from cells, assemble around Hcp tube, associate with many T6SS baseplate components, and co‐assemble with wild‐type extended sheath. This suggests that the structure of non‐contractile sheaths is very similar to the wild‐type sheath. Moreover, similarly to the wild‐type extended sheath, the non‐contractile sheaths form in the presence of ClpV and are therefore different from the previously described polysheath‐like structures, which form independently of other T6SS components but only in the absence of ClpV 11.

**Figure 5:**
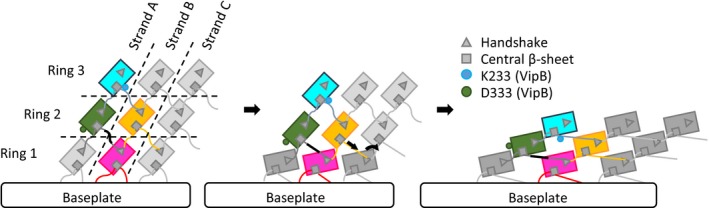
Model of the mechanism of contraction of contractile sheaths Scheme representing the connections between strands and rings in R‐type pyocin and T6SS sheaths. Ring 1 contracts after an initial trigger coming from the baseplate. Ring 1 pulls on a linker region (black) of ring 2 and by this propagates the contraction throughout the sheath. After contraction, protomers are fixed by electrostatic interactions of residue D333 of VipB. The model is depicted as viewed from the outside of the sheath.

Early electron micrographs of partially contracted T4 phage particles suggested that sheath contraction progresses in a wave of contracting sheath rings from the baseplate toward the phage head 33. However, it is currently unclear how contraction of one sheath ring triggers contraction of the next ring. As we show here, insertion of two residues into the VipA N‐terminal linker prevents T6SS sheath contraction *in vivo*, suggesting that the exact length of the linker connecting the subunits is essential for sheath contraction initiation or propagation of the contraction along the sheath (Figs 5 and EV1). In addition, T6SS, T4 phage, and R‐type pyocin sheath structures indicated charged residues potentially important for the stability of contracted structures 27, 28, 30. Many of these interactions specifically form during contraction and were thus proposed to drive sheath contraction 28, 30. Together with our data, this suggests that the newly formed interactions of VipB residue D333 stabilize the quaternary structure of the contracted state and prevent reversal of the contraction, especially when contraction is not yet completed throughout the sheath.

Interestingly, many baseplate components are enriched in the samples of the isolated stable non‐contractile sheaths as compared to the samples of contracted sheaths (Table EV2 and Dataset EV1 and Fig EV3) 1, 27. This suggests that after sheath contraction, the baseplate is destabilized and dissociates from the sheath. Live‐cell imaging of sheath dynamics and localization indeed showed that few seconds after contraction, but before disassembly, sheaths often dissociate from the initial cell envelope attachment site 16. This is consistent with the observation that T6SS assemble repeatedly inside cells and the components of the baseplate are likely reused for new rounds of assembly. In related contractile nanomachines, which are only used once, the contracted sheaths remain stably associated with the baseplates and likely provide mechanical stability to the contracted particles. In the case of contractile phages, the sheath connects baseplate and the phage head as the DNA is translocated and in the case of R‐type pyocin, the sheath might be needed to stabilize the tube, which allows ion leakage and the killing of target bacterial cell 12, 30, 35.

The approach used here to stabilize the pre‐contraction state of T6SS will likely be invaluable for further attempts to dissect T6SS mode of action at the molecular level in various bacteria and may be also used to study related contractile nanomachines with a major relevance for viral infection, bacterial competition, and pathogenicity.

## Materials and Methods

### Bacterial strains and DNA manipulations

*Vibrio cholerae* 2740‐80 parental, *vipA‐msfGFP*,* ΔvipA, ΔvipB, ΔtssE* strains and the pBAD24‐*vipA‐sfGFP* plasmid were described previously 1, 27. *vipA* mutants on pBAD24 plasmid were generated using standard techniques. Mutant *vipA* genes encode “A, AE, AEV, AEVE, AEVEL, AGAGA, AEVELP, or AEVELPL” residues inserted right after residue 25 of wild‐type *vipA*. The insertions represent either duplication of the native sequence or a sequence encoding “AGAGA”. *V. cholerae* 2740‐80 *ΔvipA*‐*vipB* strain was created by replacing *vipA* and *vipB* with a gene encoding “MSKEGSVGRLDQA” peptide (first seven residues of *vipA* and last six residues of *vipB* fused in frame) and *V. cholerae* 2740‐80 *ΔvipA‐vipB‐tssE* strain was created by replacing *vipA*,* vipB,* and *tssE* with a gene encoding “MSKEGSVRKYRVF” peptide (first seven residues of *vipA* and last six residues of *tssE* fused in frame) by allelic exchange as was done previously. *vipB* (wild type) was cloned into pBAD24 and pBAD33 plasmids using standard techniques. K223A and D333A mutations were introduced into *vipB* using mutagenic primers. All PCR‐generated products were verified by sequencing. Plasmids were transformed into *V. cholerae* by electroporation. Gentamicin‐resistant *E. coli* MG1655 strain with pUC19 plasmid was used in bacterial killing assays. Antibiotic concentrations used were streptomycin (50 μg/ml), ampicillin (200 μg/ml), chloramphenicol (20 μg/ml), and gentamicin (15 μg/ml). Lysogeny broth (LB) was used for all growth conditions. Liquid cultures were grown aerobically at 37°C.

### Bacterial killing assay

*Vibrio cholerae* 2740‐80 strains as indicated and *E. coli* MG1655 with empty pUC19 plasmid were incubated overnight at 37°C in LB supplemented with appropriate antibiotics. Cultures were diluted 100‐fold, and grown to OD 0.8–1.2 in presence of appropriate antibiotics and 0.01% arabinose for strains with pBAD plasmids (or 0.1% arabinose for sheath co‐assembly experiments). Cells were washed and mixed at final OD of ≈10 in 10:1 ratio (*V. cholerae* to *E. coli*) as specified, and 5 μl of the mixture was spotted on a pre‐dried LB agar plate containing 0.01% arabinose and ampicillin or no antibiotic. After 3 h, bacterial spots were cut out and the cells were re‐suspended in 0.5 ml LB. The cellular suspension was serially diluted (1:10) in LB, and 5 μl of the suspensions were spotted on selective plates (gentamicin for *E. coli* and streptomycin for *V. cholerae*). Colonies were counted after ≈16‐h incubation at 30°C. Two or more biological replicates were analyzed.

### Fluorescence microscopy

Procedures similar to those described previously 16 were used to detect fluorescence signal in *V. cholerae*. Overnight cultures of *V. cholerae* carrying pBAD24 plasmid with the respective inserts were diluted 100‐fold into fresh LB supplemented with ampicillin, streptomycin, and 0.01% or 0.03% arabinose and cultivated for 2.5–3.0 h to optical density (OD) at 600 nm of about 0.8–1.2. Cells from 1 ml of the culture were re‐suspended in ≈50 μl LB (to OD ≈20), spotted on a thin pad of 1% agarose in LB, and covered with a glass coverslip. For experiments in which cell lysis was induced to test the stability of VipA‐N2 sheaths, an agarose pad (1% agarose in PBS and LB 1:1) containing 0.0625× CelLytic B and 0.3 mM EDTA was used. Cells were immediately imaged at room temperature. A previously described microscope setup was used 27. VisiView software (Visitron Systems, Germany) was used to record images. Fiji 36 was used for all image analysis and manipulations as described previously 37. Bleach correction was used if necessary 38. Contrast on compared sets of images was adjusted equally. All imaging experiments were performed with three biological replicates.

### VipA/VipB sheath preparation

Overnight cultures of the indicated strains were diluted 1:1,000 in 0.5 l of fresh LB supplemented with appropriate antibiotics and then shaken at 37°C and 250 rpm to an OD of ≈1.2. Cells were centrifuged for 20 min at 5,000 *g* and 4°C, re‐suspended in 20 ml PBS, and centrifuged again for 30 min at 3,214 *g* and 4°C. Pellets were frozen until further processing. The cell pellets were thawed, re‐suspended in 20 ml of TN buffer (20 mM Tris, 150 mM NaCl, pH 8.3), and lysed by addition of (0.75×) CelLytic^™^ B, lysozyme (200 μg/ml), EDTA (5 mM), and incubation at 37°C. DNase (50 μg/ml) and MgCl_2_ (10 mM) were added to cleave DNA. After 15‐min incubation at 37°C, cell debris was removed by centrifugation for 20 min at 10,000 *g*. Cleared supernatants were subjected to ultraspeed centrifugation for 1 h at 104,000 *g* and 4°C, and the resulting pellet was washed with 1 ml TN buffer and subsequently re‐suspended in 1 ml TN buffer; insoluble material was removed by centrifugation for 1 min at 10,000 *g*. Cleared supernatants were subjected to a second round of ultraspeed centrifugation for 1 h at 104,000 *g* and 4°C, and the resulting pellet was washed with 1 ml TN buffer and subsequently re‐suspended in 1 ml TN buffer; insoluble material was removed by centrifugation for 1 min at 10,000 *g*. The supernatant was subjected to a third round of ultraspeed centrifugation for 1 h at 104,000 *g* and 4°C, and the resulting pellet was re‐suspended in 70 μl of TN buffer for further analysis. Purity of the sample was assessed by Coomassie‐stained SDS–PAGE.

### Mass spectrometry

Sheath samples (30–100 μg) were reduced with 5 mM Tris(2‐chloroethyl)phosphate, shaking for 1 h at 37°C and alkylated with 10 mM iodoacetamide, shaking for 30 min at 25°C in the dark. Proteins were digested using sequencing‐grade modified trypsin (1/250, w/w; Promega, USA) overnight at 37°C. After digestion, the samples were supplemented with trifluoroacetic acid to a final concentration of 1%. Peptides were desalted on C18 reversed‐phase spin columns according to the manufacturer's instructions (Microspin, Harvard Apparatus, Holliston, Massachusetts, USA), dried under vacuum and re‐suspended in LC‐MS buffer (0.15% formic acid, 2% acetonitrile in HPLC water) at ≈0.5 mg/ml. 1 μg of peptides of each sample was subjected to LC‐MS analysis using a dual‐pressure LTQ‐Orbitrap Elite mass spectrometer connected to an electrospray ion source (both Thermo Fisher Scientific, Waltham, Massachusetts, USA) as described recently 39 with a few modifications. In brief, peptide separation was carried out using an EASY nLC‐1000 system (Thermo Fisher Scientific, Waltham, Massachusetts, USA) equipped with a RP‐HPLC column (75 μm × 30 cm) packed in‐house with C18 resin (ReproSil‐Pur C18–AQ, 1.9 μm resin; Dr. Maisch GmbH, Ammerbuch‐Entringen, Germany) using a linear gradient from 95% solvent A (0.15% formic acid, 2% acetonitrile) and 5% solvent B (98% acetonitrile, 0.15% formic acid) to 28% solvent B over 75 min at a flow rate of 0.2 μl/min. The data acquisition mode was set to obtain one high‐resolution MS scan in the FT part of the mass spectrometer at a resolution of 240,000 full width at half maximum (at m/z 400) followed by MS/MS scans in the linear ion trap of the 20 most intense ions. The charged state screening modus was enabled to exclude unassigned and singly charged ions, and the dynamic exclusion duration was set to 20 s. The ion accumulation time was set to 300 ms (MS) and 50 ms (MS/MS). The collision energy was set to 35%, and one microscan was acquired for each spectrum. For all LC‐MS measurements, singly charged ions and ions with unassigned charge state were excluded from triggering MS2 events. MS raw files were imported into the Progenesis LC‐MS software (Nonlinear Dynamics, Version 4.0, Newcastle upon Tyne, UK) and processed using the default parameter settings. MS/MS data were exported directly from Progenesis in mgf format and analyzed using Mascot (Matrix Science, Version 2.4.0, Boston, Massachusetts, USA), searching a concatenated target‐decoy database including forward and reversed sequences of the protein entries included in the Uniprot *Vibrio cholerae* proteome database (www.uniprot.org, release date 11/07/2016, 3,784 target sequences). The Mascot search criteria were set as follows: 10 ppm precursor ion mass tolerance, 0.6 Da fragment ion mass tolerance, full tryptic specificity required (cleavage after lysine or arginine residues); maximum three missed cleavages; fixed modification: carbamidomethylation (C), variable modification: oxidation (M). Results from the database search were imported into Progenesis. The database search results were filtered, limiting the peptide and protein level FDR to 1%. The Progenesis analysis results were further processed using the SafeQuant R package 40, to obtain protein relative abundances. This analysis included summation of MS1 peak areas per protein followed by global normalization of protein abundances, per LC‐MS run, based on the abundances of proteins VipA (VC_A0107) and VipB (VC_A0108). Finally, protein abundance ratios in Table EV2 were calculated with respect to the wild‐type condition, upon averaging across biological replicates. Testing for protein differential abundance was done using empirical Bayes method 41. The resulting *P*‐values, reflecting the probability of detecting a given mean abundance difference across sample conditions by chance alone, were corrected for multiple testing using the Benjamini–Hochberg method 42.

### Statistical testing

Unpaired Student's *t*‐test was used to evaluate statistical significance of biological replicates for the bacterial killing assays and mass spectrometry.

### Negative staining electron microscopy

300‐mesh copper grids were glow‐discharged for 20 s, and samples (5 μl, protein concentration approx. 0.1 μg/ml) were adsorbed for 1 min and blotted using Whatman #1 filter paper. The grids were washed five times with ddH_2_O, and once using 2% uranyl acetate, followed by a 20‐s staining with 2% uranyl acetate. Grids were imaged on a CM‐100 microscope (Philips N.V., Amsterdam, Netherlands) equipped with a Veleta 2 k × 2 k camera (Olympus K.K., Tokyo, Japan) at 80 keV and a magnification of 64,000× (7.4 Å pixel size, 1,376 × 1,032 pixel) or a KeenView camera (Olympus K.K., Tokyo, Japan) at a magnification of 20,000× (9.1 Å pixel size) and the iTEM user interface. Fiji 36 was used for all image analysis.

### Immunoblot analysis

5–10 μl of purified sheath samples was mixed with 1.2–2.4 μl 4× NuPAGE^®^ LDS Sample Buffer (Life Technologies, Carlsbad, California, USA). Samples were incubated for 10 min at 95°C, centrifuged, cooled, and 2 μl 1 M DTT was added. Samples were heated again for 10 min at 72°C, centrifuged, loaded on 10% polyacrylamide gels, and stained with InstantBlue Protein Stain (Expedeon, San Diego, California, USA) or transferred to nitrocellulose membrane (GE Healthcare, Little Chalfont, UK). The membrane was incubated with Ponceau S (Sigma‐Aldrich, Darmstadt, Germany) to check blotting efficiency. Membrane was blocked with 5% milk in Tris‐buffered saline (pH 7.4) containing Tween 0.1% (TBST), incubated with primary peptide antibody against Hcp (“QSGQPSGQRVHKPF”, Genscript, Piscataway, New Jersey, USA 1), or peptide antibody against VipB (“QENPPADVRSRRPL”, Genscript, Piscataway, New Jersey, USA 27) for 16 h at 4°C or 1 h at room temperature, washed with TBST, incubated for 1 h with horseradish peroxidase‐labeled anti‐rabbit antibody (Jackson ImmunoResearch Inc., USA), and washed with the recommended buffer, and peroxidase was detected by LumiGLO^®^ Chemiluminescent Substrate (KPL, Inc., Gaithersburg, Maryland, USA). Nitrocellulose membrane was stripped using Restore^™^ Western Blot Stripping Buffer (Thermo Fisher Scientific, Waltham, Massachusetts, USA) and reprobed using the same protocol.

### Molecular analysis

Structures of PA0622 in the contracted state (PDB ID: 3J9R) 30 and of VipA and VipB in the contracted state (PDB ID: 3J9G) 27 were aligned based on their 3D structures using UCSF Chimera 43.

## Author contributions

MBr generated and characterized the mutant sheaths, performed fluorescence microscopy, isolated and purified the sheaths, and performed negative‐stain electron microscopy, mass spectrometry, and Western blot analysis of their structure and composition. JW contributed to electron microscopy data analysis. MBa conceived the project and analyzed the data. MBr and MBa wrote the manuscript.

## Conflict of interest

The authors declare that they have no conflict of interest.

## Supporting information



Expanded View Figures PDF

Table EV1

Table EV2

Dataset EV1

Movie EV1

Movie EV2

Movie EV3

Movie EV4

Movie EV5

Review Process File

Source Data for Figure 3
