# Supplementary material for: Type VI secretion system sheath inter‐subunit interactions modulate its contraction
Source: EMBO Rep. 2017 Dec 8;19(2):225–33. doi: 10.15252/embr.201744416 (PMC5797969; doi:10.15252/embr.201744416)
Supplement: Supplementary file 1 — Expanded View Figures PDF [file EMBR-19-225-s001.pdf]

Expanded View Figures

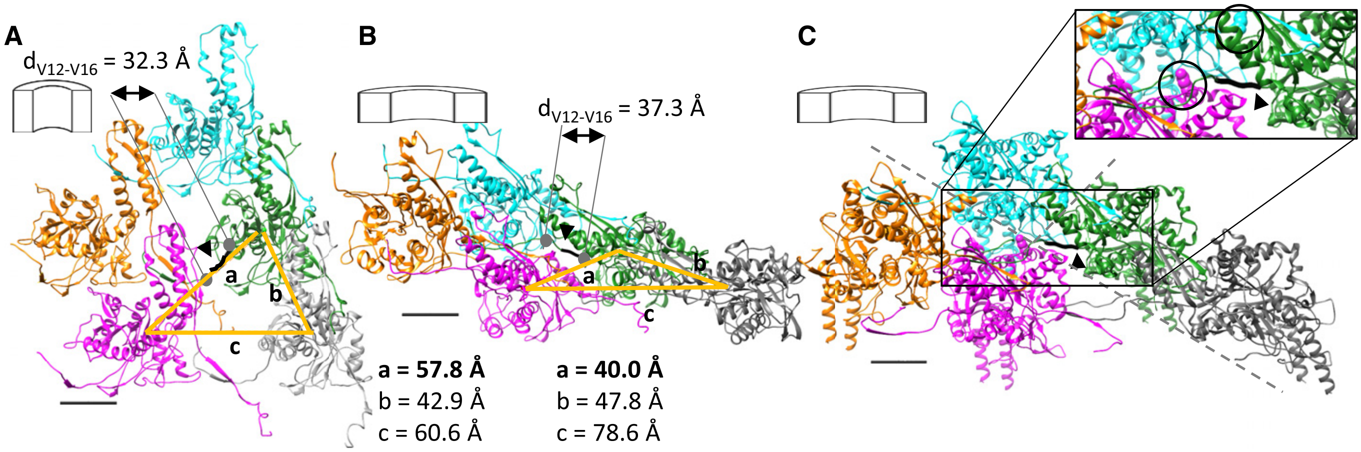

Figure EV1. Protomers of contractile sheaths are interconnected by linkers that are stretched in the contracted state.

A Structure of an R-type pyocin sheath in the extended state (PDB ID: 3J9Q) viewed from inside the sheath. Individual protomers are interlaced via linkers (black, arrowhead) through interactions formed by β-sheets.

B Structure of an R-type pyocin sheath in the contracted state (PDB ID: 3J9R). The linkers interconnecting (black, arrowhead) two strands are stretched.

C Structure of the contracted T6SS sheath from *V. cholerae* (PDB ID: 3J9G). The backbone of the linker region of VipA that connects the green protomer with the magenta protomer is shown thick in black (arrowhead). Mutated residues on VipB are highlighted in black circles in the inset. D333 is shown on the cyan protomer, and K223 is shown on the magenta protomer. Distances between center of masses of different protomers are depicted as orange lines.

Data information: The scale bars in all panels are 20 Å.

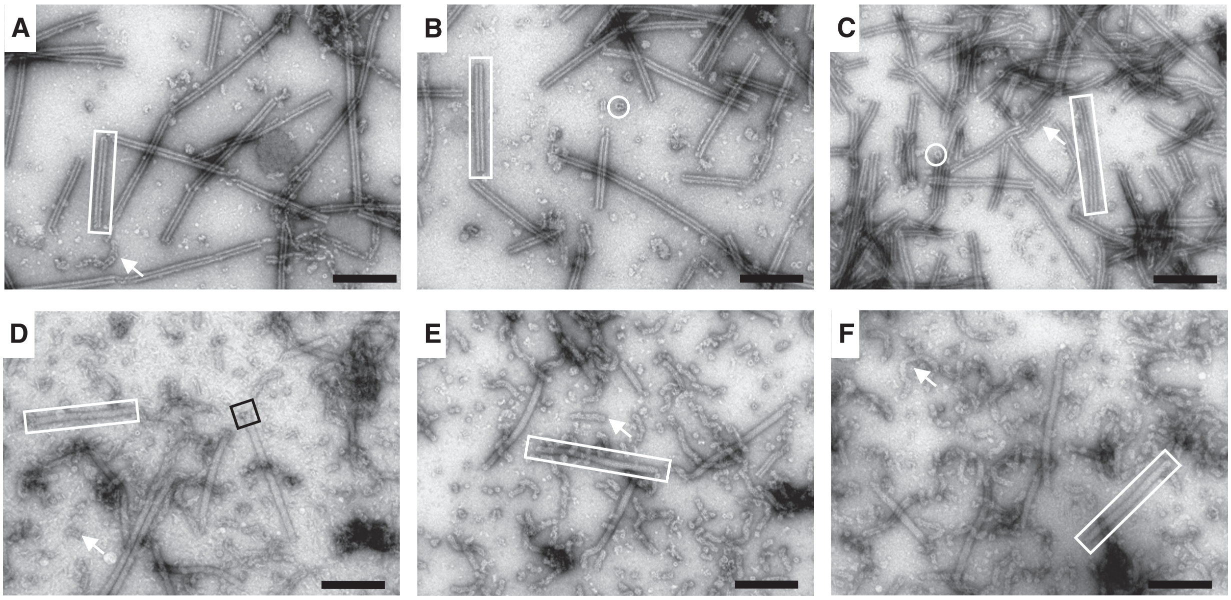

Figure EV2. Isolated sheaths appear in two different conformations.
Negative-stain electron micrographs of purified sheaths.

A–F (A) Isolated wild-type sheaths. (B) Isolated VipA-N1 sheaths. (C) Isolated VipA-N2 sheaths. (D) Isolated VipA-N3 sheaths. (E) Isolated VipA-N5 sheaths. (F) Isolated VipA-N5 (GA) sheaths. Hollow (A–C) and filled sheaths (D–F) can be observed (rectangles) as well as cogwheel-like structures (circles). Disintegrated sheaths (arrows) are observed in some cases, especially for N3, N5, and N5(GA). An example of a baseplate-like structure (black square) is highlighted. Scale bars are 200 nm.

 © 2017 The Authors

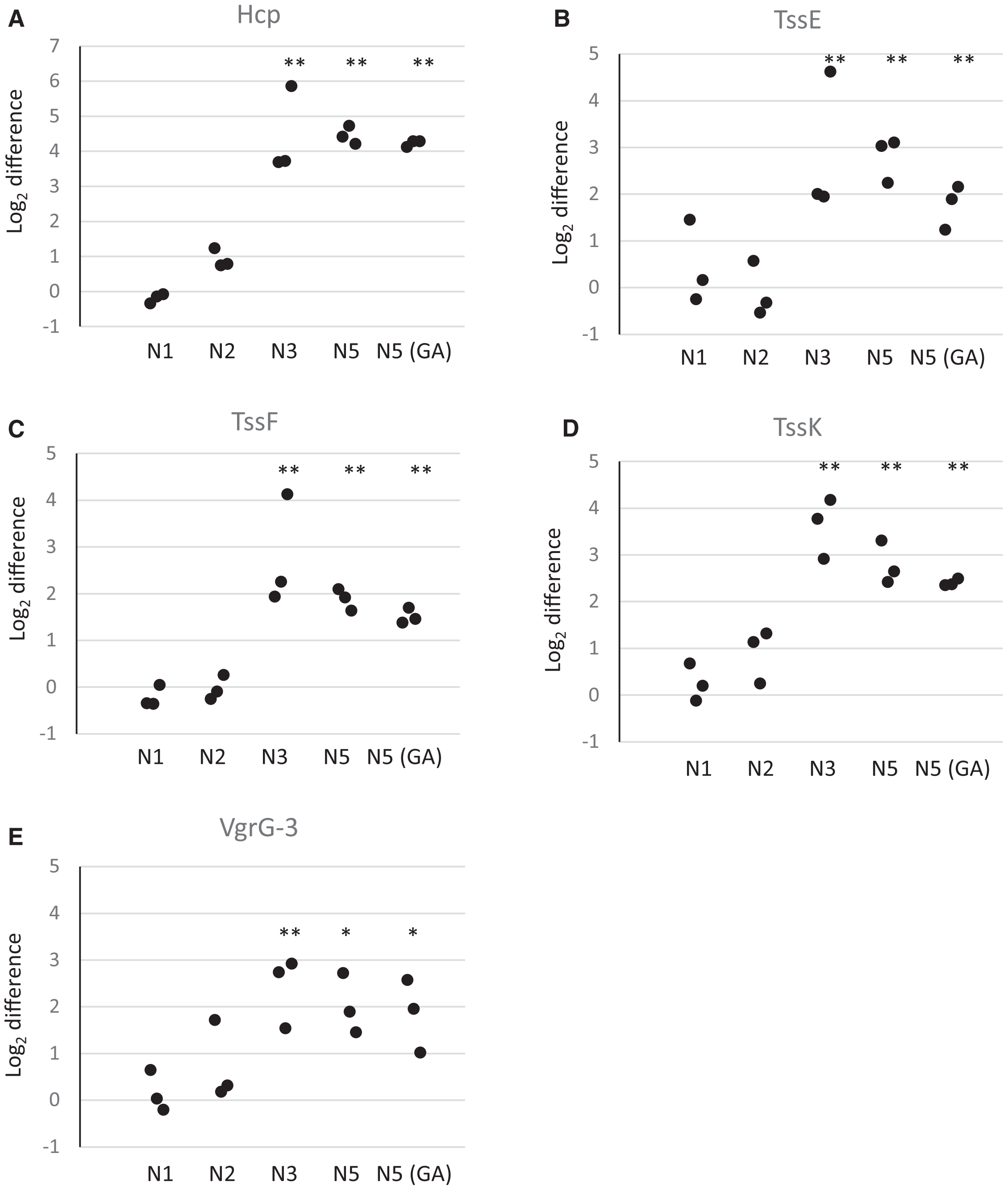

Figure EV3. The tube protein Hcp, baseplate proteins TssE, TssF, and TssK, and VgrG-3 are enriched in VipA mutants with a linker elongated by three or more amino acids.

A–E MS1 peak intensities of different T6SS-related proteins are displayed relative to wild-type protein levels. Three independent biological replicates were analyzed, and individual data points are displayed. (A) Hcp. (B) TssE. (C) TssF. (D) TssK. (E) VgrG-3. $*P \le 0.02$, $**P \le 0.001$ (Student's *t*-test).

© 2017 The Authors

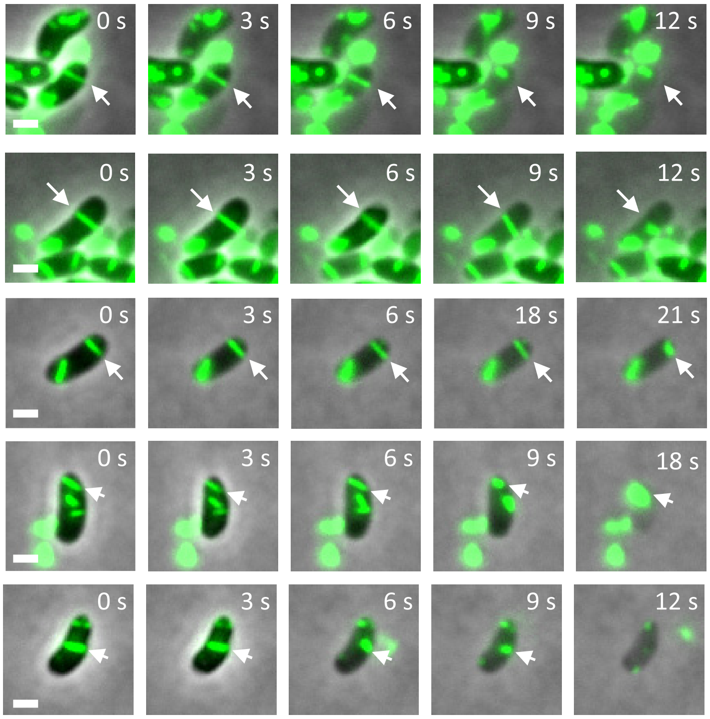

Figure EV4. VipA-N2 sheaths contract during cell lysis.

Vibrio cholerae vipA^{-} complemented with msfGFP-tagged *vipA-N2* on pBAD24 are incubated on an agarose pad containing CelLytic B and EDTA. Lysis happens within minutes, and arrows mark sheaths that contract immediately before, during, or shortly after lysis. Representative images are shown. Scale bars are 1 μm.

 © 2017 The Authors
