# Supplementary material for: Type VI secretion system sheath inter‐subunit interactions modulate its contraction
Source: EMBO Rep. 2017 Dec 8;19(2):225–33. doi: 10.15252/embr.201744416 (PMC5797969; doi:10.15252/embr.201744416)
Supplement: Supplementary file 2 — Table EV1 [file EMBR-19-225-s002.docx]

**Table EV1 - Wild type and mutant sheaths have the similar dimensions.**

For each strain, 1000 sheaths were measured.

|  | Mean length (µm) | Standard deviation (µm) | Maximum length (µm) |
| --- | --- | --- | --- |
| *V. cholerae vipA^-^*, pBAD24_vipA-msfGFP | 0.59 | 0.14 | 1.24 |
| *V. cholerae vipA^-^*, pBAD24_vipA-N2-msfGFP | 0.58 | 0.31 | 2.40 |
| *V. cholerae vipB^-^*, *vipA-msfGFP,*pBAD24_vipB | 0.55 | 0.31 | 3.15 |
| *V. cholerae vipB^-^*, *vipA-msfGFP,* pBAD24_vipB-D333A | 0.53 | 0.26 | 1.81 |
