# Supplementary material for: Type VI secretion system sheath inter‐subunit interactions modulate its contraction
Source: EMBO Rep. 2017 Dec 8;19(2):225–33. doi: 10.15252/embr.201744416 (PMC5797969; doi:10.15252/embr.201744416)
Supplement: Supplementary file 3 — Table EV2 [file EMBR-19-225-s003.docx]

**Table EV2 - Hcp, ClpV, baseplate components and the membrane complex protein TssM are detected by mass spectrometry.**

Ratio of indicated T6SS-proteins, normalized to the respective protein in wild-type sample. Average of 3 biological replicates are shown with the respective p-values (Student’s t-test). Only T6SS related proteins that were present in all three replicates are shown.

|  | contracted | | | | extended | | | | | | all extended to all contracted | |
| --- | --- | --- | --- | --- | --- | --- | --- | --- | --- | --- | --- | --- |
|  | VipA-N1 | | VipA-N2 | | VipA-N3 | | VipA-N5 | | VipA-N5 (GA) | |  |  |
|  | Log_2_ ratio | p-Value | Log_2_ ratio | p-Value | Log_2_ ratio | p-Value | Log_2_ ratio | p-Value | Log_2_ ratio | p-Value | Log_2_ ratio | p-Value |
| Hcp | -0.186 | 0.427 | 0.923 | 0.021 | 4.428 | 0.000 | 4.457 | 0.000 | 4.235 | 0.000 | 4.005 | 0.0000 |
| VgrG-3 | 0.162 | 0.569 | 0.741 | 0.118 | 2.403 | 0.001 | 2.026 | 0.001 | 1.854 | 0.001 | 1.643 | 0.0005 |
| TssM | 0.737 | 0.126 | -0.304 | 0.409 | 0.967 | 0.119 | 1.210 | 0.007 | 1.241 | 0.003 | 0.923 | 0.0330 |
| TssA1 | -0.386 | 0.354 | -0.260 | 0.496 | 0.679 | 0.222 | 0.735 | 0.176 | 0.747 | 0.029 | 1.043 | 0.0099 |
| ClpV | 0.406 | 0.356 | -0.077 | 0.822 | 0.426 | 0.421 | 1.261 | 0.008 | 0.728 | 0.106 | 0.641 | 0.0907 |
| TssK | 0.249 | 0.379 | 0.900 | 0.040 | 3.621 | 0.000 | 2.791 | 0.000 | 2.405 | 0.000 | 2.364 | 0.0000 |
| Fha | -0.329 | 0.411 | -0.572 | 0.142 | -0.172 | 0.744 | 1.345 | 0.004 | 0.175 | 0.735 | 0.900 | 0.0705 |
| TssG | -0.624 | 0.062 | 0.328 | 0.394 | 5.073 | 0.000 | 3.053 | 0.000 | 2.604 | 0.000 | 3.725 | 0.0000 |
| TssF | -0.221 | 0.373 | -0.030 | 0.920 | 2.772 | 0.000 | 1.883 | 0.001 | 1.514 | 0.001 | 2.182 | 0.0000 |
| TssE | 0.454 | 0.304 | -0.096 | 0.789 | 2.856 | 0.001 | 2.791 | 0.000 | 1.763 | 0.001 | 2.291 | 0.0003 |
