# Supplementary material for: Type VI secretion system sheath inter‐subunit interactions modulate its contraction
Source: EMBO Rep. 2017 Dec 8;19(2):225–33. doi: 10.15252/embr.201744416 (PMC5797969; doi:10.15252/embr.201744416)

Raw images – Fig. 3C – α-VipB

Chemiluminescence

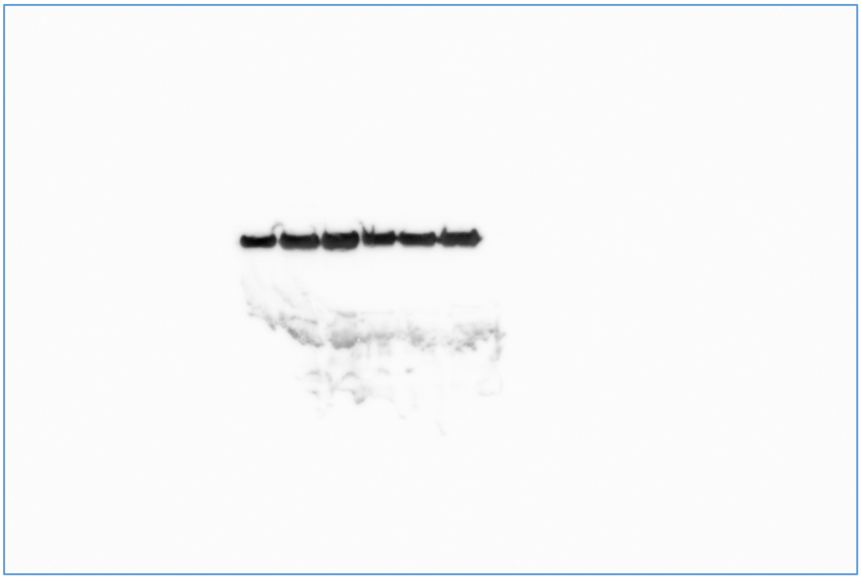

Membrane with marker

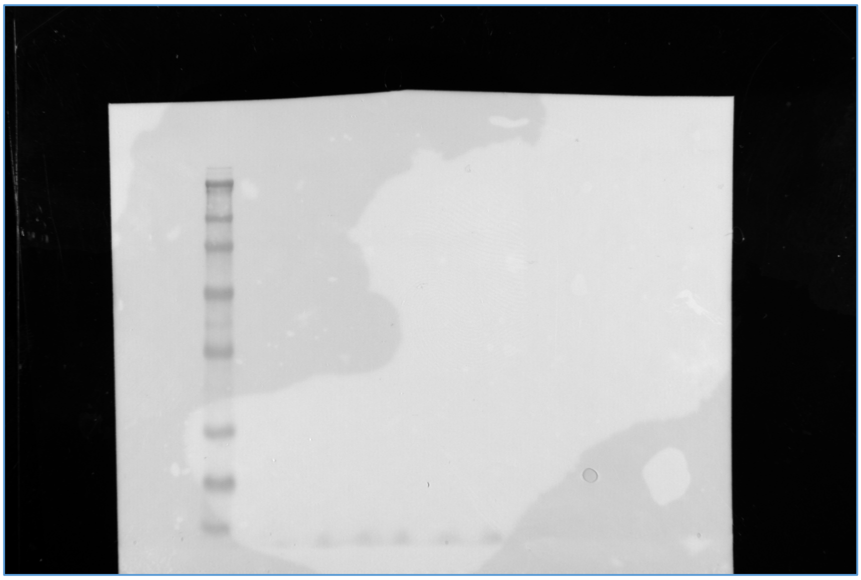

Aligned cropped images with molecular weights

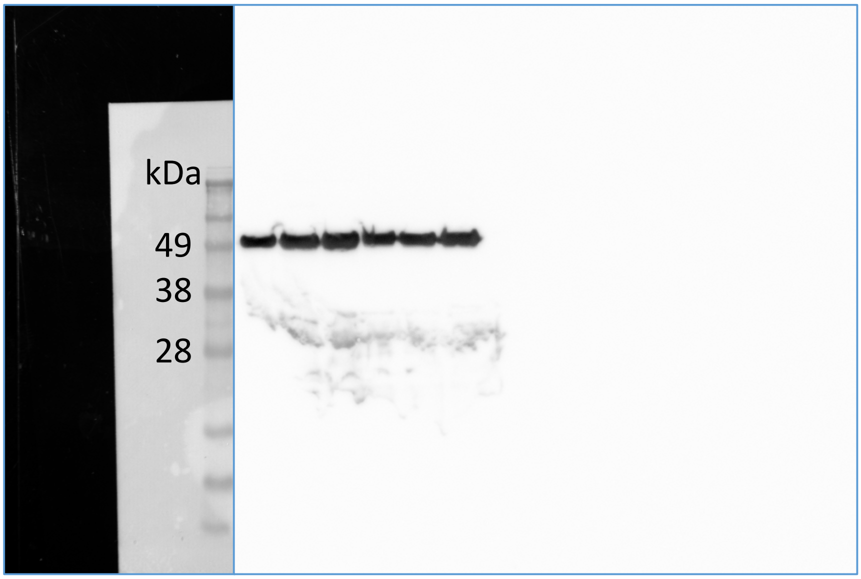

Raw images – Fig. 3C – α-Hcp

Chemiluminescence

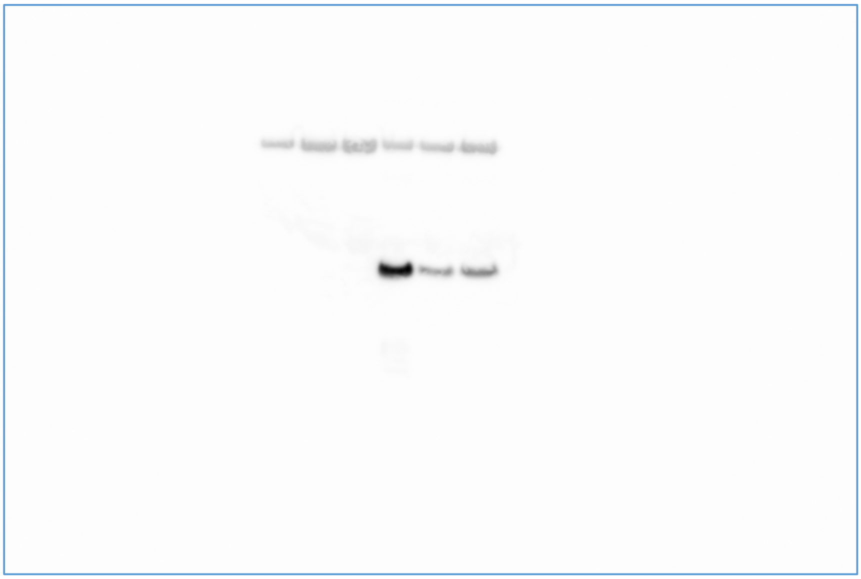

Membrane with marker

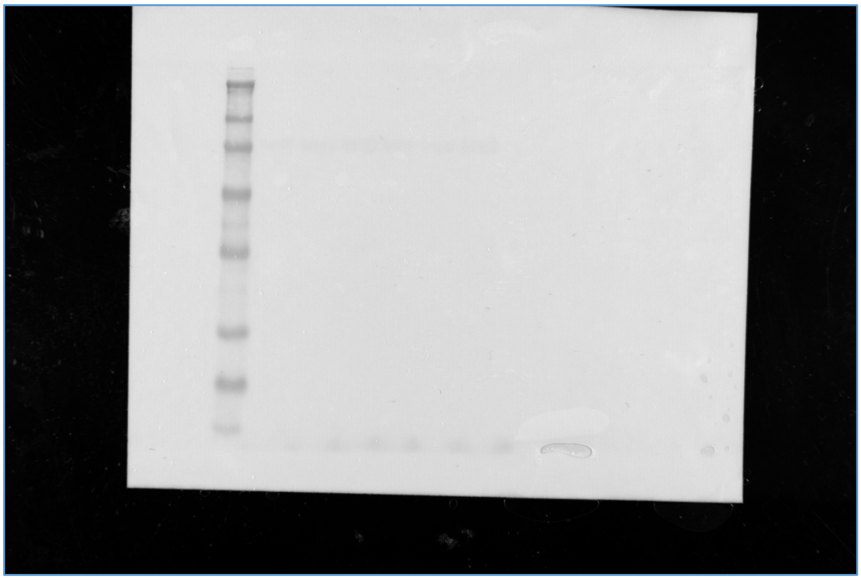

Aligned cropped images with molecular weights

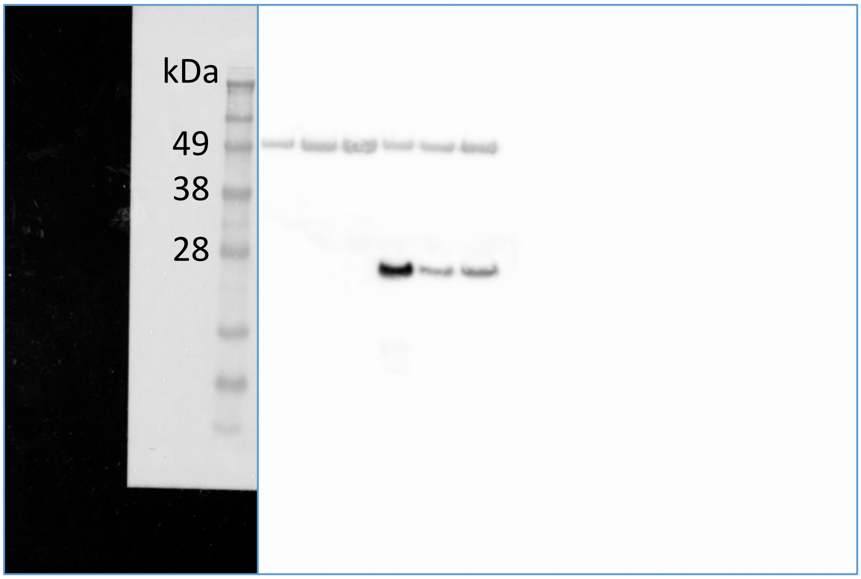

Supplement: Supplementary file 11 — Source Data for Figure 3 [file EMBR-19-225-s010.pdf]
